# Monkey Bites among US Military Members, Afghanistan, 2011

**DOI:** 10.3201/eid1904.121505

**Published:** 2013-04

**Authors:** Gregory A. Engel, Agustin Fuentes, Benjamin P.Y.-H. Lee, Michael A. Schillaci, Lisa Jones-Engel

**Affiliations:** Swedish Family Medicine, Seattle, Washington, USA (G.A. Engel);; University of Notre Dame, South Bend, Indiana, USA (A. Fuentes);; National Parks Board, Singapore (B.P.Y.-H. Lee);; University of Toronto Scarborough, Toronto, Ontario, Canada (M.A. Schillaci);; University of Washington, Seattle (G.A. Engel, L. Jones-Engel)

**Keywords:** Macaca mulatta, monkey bites, rabies, herpes B virus, viruses, zoonoses, US military, Afghanistan

**To the Editor:** We take serious issue with the dispatch by Mease and Baker on monkey bites among US military members in Afghanistan during 2011 (*1*). In particular, we are troubled by the first paragraph. The dispatch opens by listing bites from rhesus macaques (*Macaca mulatta*) as one of the many risks faced by military personnel deployed to Afghanistan. Although technically a true statement, it is misleading in its perspective. Since 2001, ≈2,000 US soldiers have died in Afghanistan and another ≈18,000 have been wounded in action (*2*). The authors juxtapose this toll with minor injuries incurred by 10 soldiers who flouted explicit rules prohibiting contact with pet monkeys.

None of the bitten soldiers were reported to have sequelae. Furthermore, the first paragraph leaves the impression that a US Army soldier who died of rabies while serving in eastern Afghanistan may have contracted the disease from a macaque. This finding would be an extremely unlikely occurrence.

We have yet to see a single credible report of macaque-to-human transmission of rabies. In fact, we have yet to see a report of naturally acquired rabies infection in a macaque. Similarly, although antiviral prophylaxis is routinely prescribed to persons bitten by rhesus monkeys, there is not a single report of herpes B virus infection in a human outside the laboratory/zoo context, although thousands of persons are likely bitten by macaques in Asia every year (*3*,*4*).

In contrast, zoonotic transmission of simian foamy virus, a retrovirus ubiquitous in nonhuman primates, has been shown to occur from macaques to humans, probably through monkey bites, although this virus has not been shown to cause disease in humans (*5*). Although it is advisable to avoid contact with monkeys, risk for disease transmission should be placed in proper perspective. Exaggerating risks of bites has, in the past, led to irrational culling of entire populations of macaques (*6*).

## References

[1] Mease LE, Baker KA. Monkey bites among US military members, Afghanistan, 2011. Emerg Infect Dis. 2012;18:1647–9. 10.3201/eid1810.120419 23017939 PMC3471630

[2] US Department of Defense. Casualty status: Operation Iraqi Freedom; Operation New Dawn; Operation Enduring Freedom. November 6, 2012 [cited 2012 Nov 7]. http://www.defense.gov/news/casualty.pdf

[3] Engel GA, Jones-Engel L, Schillaci MA, Suaryana KG, Putra A, Fuentes A. Human exposure to herpesvirus B–seropositive macaques, Bali, Indonesia. Emerg Infect Dis. 2002;8:789–95. 10.3201/eid0808.010467 12141963 PMC3266706

[4] Fuentes A, Gamerl S. Disproportionate participation by age/sex classes in aggressive interactions between long-tailed macaques (*Macaca fascicularis*) and human tourists at Padangtegal Monkey Forest, Bali, Indonesia. Am J Primatol. 2005;66:197–204. 10.1002/ajp.20138 15940713

[5] Jones-Engel L, Engel GA, Schillaci MA, Aida Rompis A, Putra A, Suaryana KG, Primate-to-human retroviral transmission in Asia. Emerg Infect Dis. 2005;11:1028–35. 10.3201/eid1107.040957 16022776 PMC3371821

[6] Monkeys with herpes B virus culled at a safari park. Commun Dis Rep CDR Wkly. 2000;10:99,102. 10769489

