# Supplementary material for: Monkey Bites among US Military Members, Afghanistan, 2011
Source: Emerg Infect Dis. 2013 Apr;19(4):691–2. doi: 10.3201/eid1904.121505 (PMC5836485; doi:10.3201/eid1904.121505)
Supplement: Related texts

# Reply: Monkey Bites among U.S. Military Members, Afghanistan, 2011

**Authors:** Luke E. Mease, Katheryn A. Baker

**Affiliations:** Army Health Clinic, Dugway Proving Ground, Utah, USA (L.E. Mease);; General Leonard Wood Army Community Hospital, Fort Leonard Wood, Missouri, USA (K.A. Baker)

**In Response:** In response to the letter by Engel et al. (*1*), we concur that combat-related deaths and illness are a greater risk than monkey bites for deployed military personnel. Furthermore, we agree that risk for monkey bites should be considered in perspective with other risks faced by deployed personnel. We also believe that action taken to decrease macaque populations in response to risks mentioned would be irrational and inappropriate; in a country affected by war, wildlife conservation efforts are needed. We did not intend to imply that the rabies-associated death mentioned in our article was caused by contact with a macaque (*2*). As reported elsewhere, the patient likely contracted rabies from a dog bite (*3*).

Nonetheless, we believe that risk for monkey bites deserves the attention of deployed medical providers. Risks for bacterial infection and major local trauma are critical for any macaque bite. We acknowledge that risk for contracting viral disease (rabies or B virus infection) from macaques in the wild is probably low, but we believe that it merits consideration.

Social conditions in Afghanistan have prevented any substantial rabies prevention program. Consequently, prevalence of rabies among wild animals and pets is unknown and could be higher than in other countries. Absence of any documented human deaths from B virus in a country with a developing medical system and a high mortality rate does not confirm absence of risk. B virus has been shown to be fatal in other areas, particularly in countries with greater medical diagnostic capacity (*4*).

Engel et al. suggest that recorded monkey bites occurred because affected persons flouted rules prohibiting contact with local animals. Given the unpredictable nature of operations in Afghanistan, it is impossible to determine fault for the animal bites detailed. Furthermore, blaming bite victims may be counterproductive, exacerbating underreporting and discouraging deployed personnel from seeking needed care. We believe that the role of command support and responsibility cannot be overemphasized in preventing deployed personnel from interacting with local animals (*5*). We thank Engel et al. for providing additional perspective on the risk for monkey bites to personnel deployed in Afghanistan.

## References

[1] Engel GA, Fuentes A, Lee BPY-H, Schillaci MA, Jones-Engel L. Monkey bites among US military members, Afghanistan, 2011. Emerg Infect Dis. 2013;19:691.10.3201/eid1904.121505PMC583648523750611

[2] Mease LE, Baker KA. Monkey bites among US military members, Afghanistan, 2011. Emerg Infect Dis. 2012;18:1647–9. 10.3201/eid1810.12041923017939PMC3471630

[3] Centers for Disease Control and Prevention. Imported human rabies in a U.S. Army soldier—New York, 2011. MMWR Morb Mortal Wkly Rep. 2012;61:302–5 .22552206

[4] Huff JL, Barry PA. B-virus (Cercopithecine herpesvirus 1) infection in humans and macaques: potential for zoonotic disease. Emerg Infect Dis. 2003;9:246–50. 10.3201/eid0902.02027212603998PMC2901951

[5] Chretien JP. Protecting service members in war–non-battle morbidity and command responsibility. N Engl J Med. 2012;366:677–9. 10.1056/NEJMp111298122356320
